# Protein S-Nitrosylation in Heart Failure: A Compartment-Resolved Review of Mechanisms, Evidence Boundaries, and Translational Perspectives

**DOI:** 10.3390/antiox15060716

**Published:** 2026-06-04

**Authors:** Miao Shi, Yongnan Li, Ziwei Zhu, Yafei Xie, Xiaowei Zhang

**Affiliations:** 1 Department of Cardiology, Lanzhou University Second Hospital, Lanzhou University, Lanzhou 730000, China; shmiao2024@lzu.edu.cn (M.S.);; 2 Department of Cardiac Surgery, Lanzhou University Second Hospital, Lanzhou 730031, China

**Keywords:** S-nitrosylation, heart failure, heart failure with preserved ejection fraction, nitric oxide, nitrosative stress

## Abstract

Heart failure (HF) remains a major cause of morbidity and mortality despite substantial therapeutic progress, and important phenotype-specific treatment gaps persist. Protein S-nitrosylation (SNO) is a reversible cysteine-centered post-translational modification (PTM) whose reported associations with selected HF-relevant contexts, including vascular–endothelial dysfunction, mitochondrial–energetic remodeling, Ca^2+^-handling abnormalities, and selected receptor- or stress-related signaling observations, are supported to varying degrees. In this review, we evaluate reported mechanisms that may regulate cardiac SNO and define the evidentiary boundaries that constrain interpretation across HF-relevant settings. Available studies suggest that altered SNO homeostasis is associated with selected HF-related processes, but the strength of support varies substantially across targets, phenotypes, and disease contexts. Many mechanistic observations derive from animal models, cultured systems, donor-based perturbations, or non-HF settings. These should, therefore, be interpreted as hypothesis-generating rather than as established mechanisms in human HF. We accordingly distinguish findings supported by human HF tissue or HF-relevant in vivo evidence from more preliminary observations and highlight the need for human, site-resolved, and, where feasible, quantitatively grounded datasets. Future studies should prioritize stronger tissue anchoring, better integration of circulating and myocardial readouts, and closer alignment between mechanistic claims and the strength of the supporting evidence.

## 1. Introduction

Heart failure (HF) remains a leading cause of morbidity and mortality worldwide and continues to impose substantial residual risk despite expanding guideline-directed therapies [1,2]. Contemporary randomized trials have expanded the evidence base for therapy across HF phenotypes, including the demonstrated benefit of sodium–glucose cotransporter 2 (SGLT2) inhibitors in heart failure with mildly reduced ejection fraction (HFmrEF) and heart failure with preserved ejection fraction (HFpEF) [3,4,5]. Nevertheless, many patients continue to experience persistent symptoms and recurrent hospitalizations, underscoring important phenotype-specific treatment gaps and motivating further examination of regulatory processes relevant to HF pathophysiology [1,6].

Protein post-translational modifications (PTMs) can reflect context- and phenotype-dependent signaling states in cardiovascular disease [7,8,9]. Among cysteine-centered PTMs, S-nitrosylation (SNO) is a reversible modification that has been reported to affect protein function under specific cellular conditions. Changes in SNO should not be assumed to parallel changes in canonical nitric oxide (NO)–soluble guanylate cyclase (sGC)–cyclic guanosine monophosphate (cGMP)–protein kinase G (PKG) signaling [10,11,12,13,14,15]. Although this review focuses on SNO, its effects should be interpreted in the context of other cysteine-centered modifications, including S-glutathionylation, sulfenylation, disulfide formation, and irreversible oxidation under sustained stress.

**Figure 1:**
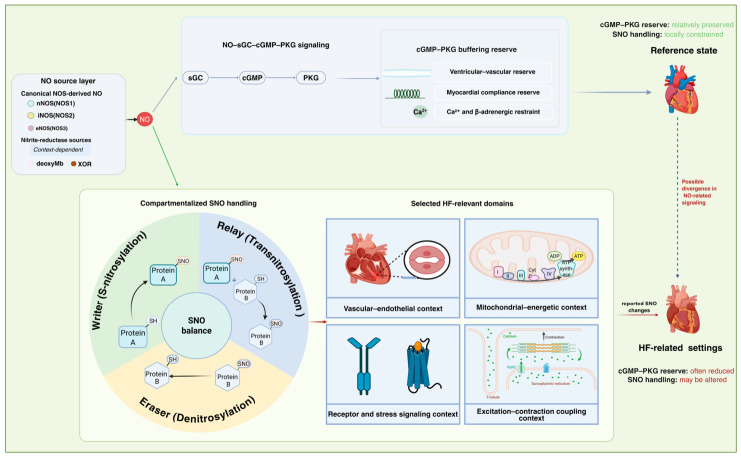
Dual-layer NO-related signaling framework for interpreting S-nitrosylation in HF-related settings. Schematic overview of NO-related signaling layers discussed in this review. The source layer includes canonical NOS-derived NO from nNOS, iNOS, and eNOS, together with context-dependent nitrite-reductase inputs, illustrated here by deoxymyoglobin (deoxyMb) and xanthine oxidoreductase (XOR). The upper pathway illustrates NO–sGC–cGMP–PKG signaling and related system-level buffering reserve. The lower pathway illustrates compartmentalized SNO handling, including source input, transnitrosylation, and denitrosylation, together with selected HF-relevant contexts discussed in later sections. The right-side comparison is intended as an illustrative contrast between a reference state, characterized by relatively preserved cGMP–PKG reserve and locally constrained SNO handling, and selected HF-related remodeling settings, in which reduced cGMP–PKG reserve may coexist with altered SNO-related patterns. The figure is conceptual and does not imply equivalent evidence depth, causal validation, or generalizability across all HF phenotypes. The different arrow colors are used only to visually distinguish schematic pathways and do not indicate evidence level, relative pathway strength, or mechanistic hierarchy. Created in BioRender. Shi, M. (2026) https://BioRender.com/8vd042g (accessed on 21 May 2026). Abbreviations: HF, heart failure; NO, nitric oxide; NOS, nitric oxide synthase; nNOS, neuronal NOS; iNOS, inducible NOS; eNOS, endothelial NOS; deoxyMb, deoxygenated myoglobin; XOR, xanthine oxidoreductase; sGC, soluble guanylate cyclase; cGMP, cyclic guanosine monophosphate; PKG, protein kinase G; SNO, S-nitrosylation.

In this review, we discuss several considerations relevant to the interpretation of cardiac SNO, including the NO source and microdomain, local cysteine reactivity, and denitrosylation capacity over time. We then summarize current findings on altered SNO across selected HF-relevant contexts, including microvascular/endothelial dysfunction, mitochondrial–energetic remodeling, Ca^2+^-handling abnormalities, and selected receptor- or stress-related signaling observations. Figure 1 provides a conceptual overview of the relationship between canonical NO–sGC–cGMP–PKG signaling and compartmentalized SNO regulation discussed in later sections.

To keep mechanistic interpretation proportional to the available evidence, we distinguish conceptual organization from disease-validated causality throughout the review. Section 2 and Section 3 define the mechanistic and analytical framework for interpreting SNO signaling, whereas Section 4 and Section 5 separately evaluate HF-relevant evidence and translational perspectives. This structure is intended to clarify which SNO-linked mechanisms are supported by human HF or HF-relevant in vivo evidence and which should presently be regarded as hypothesis-generating.

## 2. Molecular Basis of Protein S-Nitrosylation

This section outlines the main concepts used later to interpret NO-derived signaling in HF. We distinguish diffusible NO–sGC–cGMP–PKG signaling from more locally constrained protein S-nitrosylation and then introduce selected carrier and enzymatic systems that may influence local SNO handling. Together, these concepts provide the mechanistic vocabulary for the evidence-graded examples discussed below.

### 2.1. NO-Related Signaling in the Heart

NO-related signaling in the heart can be discussed in two partly overlapping forms. The canonical NO–sGC–cGMP–PKG axis acts as a diffusible second-messenger pathway that supports hemodynamic and relaxation reserve and remains clinically relevant in heart failure with reduced ejection fraction (HFrEF) [14,16,17]. By contrast, protein S-nitrosylation is usually more local. It involves reversible modification of reactive cysteine thiols and may alter protein activity or interactions within specific cellular microdomains [10,18].

The NO–sGC–cGMP–PKG axis and protein S-nitrosylation should not be assumed to track together. In HF-related settings, reduced cGMP–PKG reserve and altered compartmental SNO have both been observed, but a direct link between them has not been established [19,20]. In the following subsections, source input, transnitrosylation, and denitrosylation are used only as practical categories for discussing local SNO handling.

### 2.2. RSNO Pools, Nitrite, and Local NO Signaling

Low-molecular-weight S-nitrosothiols (RSNOs), especially S-nitrosoglutathione (GSNO) and S-nitrosylated coenzyme A (SNO-CoA), can move nitrosylating equivalents beyond their site of formation [21,22,23]. Through thiol exchange, they may extend NO-related signaling beyond local nitric oxide synthase (NOS) microdomains. In HF-relevant settings, RSNO availability may affect how readily nitrosylation occurs, whereas local enzymatic control is more likely to influence where those signals persist or are removed [21,22,24]. RSNO formation depends strongly on chemical context. In biological environments, RSNOs can arise through oxidation-dependent nitrosating reactions, radical-mediated thiol chemistry, and metal-centered NO reactions that favor RS–NO bond formation or cleavage [22,25,26,27]. The balance among these routes varies with oxygen availability, local redox conditions, thiol buffering, and nearby metalloproteins. Because these factors can shift in failing hearts, RSNO-related measurements are difficult to interpret on their own and should not be treated as direct readouts of site-resolved protein SNO [27].

Nitrite is not part of the RSNO pool, but under hypoxic, acidic, or ischemic conditions it may provide an additional source of NO bioactivity in the heart and vessel wall [28,29]. In cardiac ischemia–reperfusion settings, nitrite can contribute not only to NO formation but also to myocardial S-nitrosothiol formation [30]. Relevant nitrite-reductase systems are noted in Section 2.3.1. RSNO pool behavior may also vary with carrier turnover, eraser activity, and conditional upstream source input.

**Figure 2:**
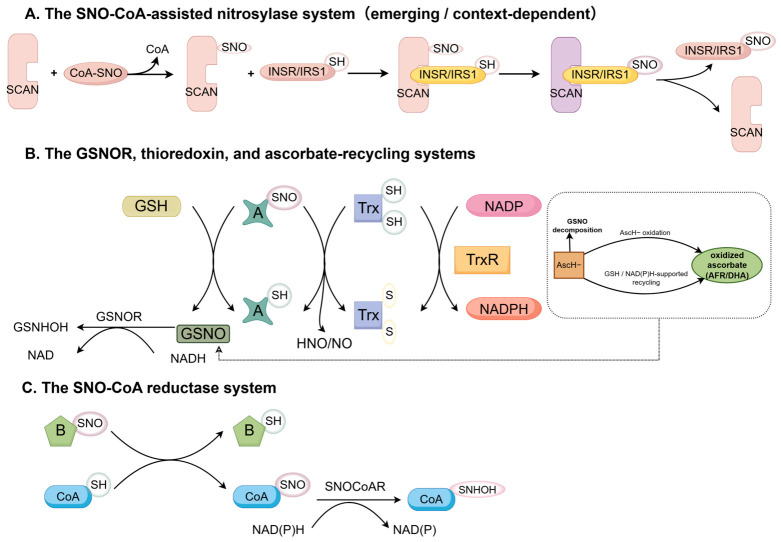
Selected enzymatic systems discussed in relation to local RSNO handling. Schematic summary of selected systems discussed in Section 2.2 and Section 2.3. The figure is intended as a conceptual aid to illustrate representative biochemical links between low-molecular-weight S-nitrosothiol pools and protein S-nitrosylation, without implying equivalent evidence depth across all systems. The dashed box denotes the ascorbate-recycling module, and dashed arrows indicate indirect recycling or reducing-equivalent connections rather than direct protein-targeting reactions. (**A**) SNO-CoA-assisted nitrosylase (SCAN) is shown as an emerging, context-dependent example through which SNO-CoA-derived nitrosylating equivalents may be transferred to protein thiols. INSR/IRS1 is included only as a representative target example. (**B**) S-nitrosoglutathione reductase (GSNOR), the thioredoxin/thioredoxin reductase (Trx/TrxR) system, and an ascorbate-recycling arm are shown as systems that can influence GSNO persistence and protein-SNO turnover. GSNOR metabolizes GSNO, the Trx/TrxR system restores reduced protein thiols, and oxidized ascorbate species (AFR/DHA) are recycled through glutathione- and NAD(P)H-dependent pathways. The ascorbate-recycling arm is shown as a modifier of GSNO availability rather than as a protein-selective denitrosylase. (**C**) SNO-CoA reductase (SCoR/AKR1A1; also known as SNOCoAR) is shown as a system that reduces SNO-CoA and can thereby shape the availability of the SNO-CoA carrier pool. By Figdraw (https://www.figdraw.com). Export ID: ISAWI1511a. Abbreviations: RSNO, S-nitrosothiol; SNO, S-nitrosylation; GSNO, S-nitrosoglutathione; GSNOR, S-nitrosoglutathione reductase; SCAN, SNO-CoA-assisted nitrosylase; SNO-CoA, S-nitrosylated coenzyme A; CoA, coenzyme A; Trx/TrxR, thioredoxin/thioredoxin reductase; GSH, glutathione; AFR, ascorbyl free radical; DHA, dehydroascorbate; SCoR/SNOCoAR, SNO-CoA reductase; INSR/IRS1, insulin receptor/insulin receptor substrate 1; NAD, nicotinamide adenine dinucleotide; NADH, reduced nicotinamide adenine dinucleotide; NADP, nicotinamide adenine dinucleotide phosphate; NADPH, reduced nicotinamide adenine dinucleotide phosphate; NAD(P)H, NADH or NADPH.

RSNO levels are influenced not only by formation but also by turnover. S-nitrosoglutathione reductase (GSNOR) and thioredoxin (Trx)-related systems affect the persistence of GSNO and protein SNO [31]. Ascorbate can further accelerate GSNO breakdown, although its overall effect depends on recycling of oxidized ascorbate through glutathione- and NAD(P)H-dependent pathways, where NAD(P)H denotes NADH or NADPH [32,33,34,35,36]. Changes in RSNO levels are therefore difficult to interpret in isolation. Without compartment-resolved quantification and matched thiol-redox context, RSNO- or nitrite-linked signals should not be taken as direct evidence of specific site-resolved protein SNO. Selected examples of source input, transnitrosylation, and denitrosylation are outlined below and summarized in Figure 2.

### 2.3. Selected Systems Influencing Cardiac S-Nitrosylation

In the heart, protein S-nitrosylation depends not only on overall NO production, but also on where NO-related species are generated, how SNO is transferred to target cysteines, and how quickly these modifications are removed. This section discusses these processes as source input, transnitrosylation, and denitrosylation in the cardiac setting. Table 1 lists selected regulators and upstream inputs relevant to these processes and groups them by functional association, without implying disease causality.

#### 2.3.1. Source Inputs in the Cardiac Setting

Enzymatic sources that may influence local NO/RSNO availability are referred to here, for convenience, as “writers.” This designation is best supported when source localization can be considered together with evidence of target engagement, rather than inferred from bulk NO-derived readouts alone. In the heart, the best-established writer tier still comprises the canonical NOS isoforms. In cardiomyocytes, neuronal nitric oxide synthase (nNOS) is enriched in junctional sarcoplasmic reticulum (SR)/excitation–contraction coupling (ECC) microdomains and may influence SNO on nearby ECC-related proteins, including ryanodine receptor 2 (RyR2) [19,37]. In parallel, endothelial nitric oxide synthase (eNOS) is a major endothelial source of NO in the coronary microvasculature and contributes to local NO bioavailability [38,39]. Its relevance to HFpEF is considered later in the context of microvascular dysfunction, rather than inferred here as a single defined mechanism [20,40]. By contrast, inducible nitric oxide synthase (iNOS) is stress-inducible. In HFpEF-related experimental systems and limited human studies, it has been linked to broader stress-associated SNO changes [41,42]. Beyond the canonical NOS isoforms, nitrite can provide an additional source of NO bioactivity under hypoxic or acidic conditions [29]. In the cardiovascular setting, deoxygenated myoglobin is the nitrite-reductase system most directly relevant to the heart, especially during myocardial ischemia–reperfusion [43,44]. Xanthine oxidoreductase provides an additional enzymatic route for nitrite reduction in hypoxic vascular and tissue compartments [45]. Under hypoxic or redox-stressed conditions, these proteins may act as noncanonical NO sources. Because direct evidence linking nitrite-reductase pathways to chronic HF-related SNO remodeling remains limited, they are considered here as conditional contributors to NO/SNO supply rather than established regulators of chronic HF SNO remodeling.

#### 2.3.2. Selected Transnitrosylation Examples in the Cardiac Setting

Here we briefly note examples in which transnitrosylation has been invoked to explain transfer of NO-related equivalents from low-molecular-weight RSNO pools or donor proteins to acceptor cysteines. This interpretation is best supported when the donor source, the acceptor target, and an associated change in target SNO can all be assessed, rather than inferred from global shifts in RSNO pools alone [46].

GAPDH is one often-cited example, because SNO has been linked to its nuclear trafficking and to downstream nuclear SNO events [47]. SCAN (SNO-CoA-assisted nitrosylase; Figure 2A) provides a second, metabolite-linked example in which an SNO-CoA pool has been proposed to contribute to protein SNO [48]. By contrast, ascorbate–glutathione chemistry is discussed here only in relation to GSNO stability and the availability of low-molecular-weight NO equivalents, not as a protein-selective relay [33,36,49]. Bulk RSNO changes alone do not identify specific downstream targets. In HF-related settings, more persuasive interpretation comes from cardiac studies that link directional SNO changes to local functional effects.

#### 2.3.3. Denitrosylation and Related Systems

This subsection focuses on systems that remove protein SNO or limit the persistence of low-molecular-weight RSNOs. Thioredoxin/thioredoxin reductase (Trx/TrxR) is a well-described protein-directed denitrosylation system and acts in both cytosolic and mitochondrial compartments [24]. GSNOR primarily metabolizes GSNO and can thereby shape GSNO-dependent transnitrosylation. In cardiac settings, reported compartment-specific GSNOR activity, including mitochondrial localization, has been associated with mitochondrial redox changes and bioenergetic stress responses [50]. Ascorbate affects this system indirectly through GSNO stability. Although it can accelerate GSNO breakdown, the net effect depends on recycling of oxidized ascorbate species such as the ascorbyl free radical (AFR) and dehydroascorbate (DHA) through GSH- and NAD(P)H-dependent pathways [32,34,51,52]. In this context, ascorbate is relevant mainly because it affects GSNO availability, not because it acts as a protein-selective denitrosylase. SNO-CoA reductase (SCoR) metabolizes SNO-CoA and may also influence the amount of this carrier available for downstream transfer reactions [23,53].

In HF-related settings, some studies have linked altered remodeling to reduced or poorly timed denitrosylation, although changes in upstream NO/SNO supply may also be involved. Most cardiac evidence remains indirect and is based on bulk tissue measurements.

### 2.4. Interpreting Local SNO in HF-Related Settings

Available evidence suggests that local control of SNO is altered in some HF-relevant settings, although direct microdomain-resolved data, especially from human myocardium, remain limited. Simply increasing NO equivalents should therefore not be assumed to restore local SNO signaling. Reported SNO changes also need to be considered alongside competing cysteine modifications, because their significance may vary with the modified cysteine, its subcellular location, and local redox conditions.

### 2.5. Competing Cysteine Modifications

S-nitrosylation occurs within a broader cysteine redox landscape rather than as an isolated modification. Reactive cysteines can also undergo S-glutathionylation, sulfenylation, disulfide formation, and, under sustained oxidative burden, progression toward less reversible oxidative states [54,55,56,57]. The functional consequence attributed to a given SNO event may reflect not only SNO occupancy itself, but also competition, sequence, or interconversion among co-existing cysteine modifications [15,54,56]. This issue is especially relevant in HF, where persistent oxidative stress, altered glutathione buffering, and compartment-specific NO/ROS interactions can change which thiol state is functionally dominant.

RyR2 provides a useful example of this issue. Reactive oxygen and nitrogen species can promote endogenous RyR2 S-nitrosylation and S-glutathionylation [58]. In an HF-relevant rat model, nitroso-redox imbalance has been linked to RyR2 oxidation, reduced RyR2 S-nitrosylation, and diastolic sarcoplasmic reticulum Ca^2+^ leak [59]. This finding is better understood within a broader redox/PTM context than as an isolated change in SNO alone.

A related caution applies to mitochondrial energetic proteins. In dyssynchronous HF, ATP synthase α-subunit Cys294 was shown to carry multiple oxidative PTMs, including disulfide formation, S-glutathionylation, and S-nitrosylation, supporting the view that this residue behaves as a redox-sensitive integrator rather than as a unidirectional SNO switch [60]. Where competing thiol states have not been resolved, interpretation should remain cautious.

## 3. Analytical Rigor and Attribution Boundaries for Protein S-Nitrosylation in HF

Because SNO studies in HF vary substantially in analytical resolution, this section outlines the level of interpretation that different readouts can reasonably support at the protein, site, and, where feasible, occupancy level [61,62]. In the HF SNO literature, residue-level wording is often applied to protein-level or system-level readouts, creating a mismatch between assay resolution and the language used to describe the findings. Endogenous SNO can be low in occupancy, chemically labile, and sensitive to the surrounding redox environment. For this reason, site-resolved identification should be distinguished from broader protein-level or system-level signals when interpreting these datasets [63].

### 3.1. Analytical Classes (A–C) and the Level of Attribution They Support

For interpretive clarity, we group commonly used SNO detection approaches into three analytical classes (A–C) based on the highest level of molecular information they typically provide: residue level (Class A), protein level (Class B), and system level (Class C). These classes are intended to define attribution limits rather than to rank methods by overall quality.

Class A includes site-resolved mass spectrometry (MS) workflows. When site localization is secure and switch-chemistry specificity has been adequately controlled, these methods can support residue-level wording. In some designs, they may also provide limited information relevant to occupancy [64]. Class B includes protein-level enrichment readouts and comparative proteome-scale findings. These approaches are useful for identifying candidate proteins but are usually semi-quantitative and do not, on their own, establish site assignment or occupancy. Class C includes scalable systemic or bulk RSNO/NO readouts, often measured in biofluids. These approaches can support phenotyping and longitudinal tracking, but they do not define protein- or site-resolved myocardial SNO without paired molecular data. Table 2 summarizes representative workflows and the corresponding writing ceiling for each class.

### 3.2. Class A and B: Site- and Protein-Level Readouts

#### 3.2.1. Site-Resolved Mass Spectrometry (Class A)

IodoTMT-Switch LC–MS/MS and Related Site-Resolved Workflows

Switch-based site-mapping workflows (e.g., iodoTMT/iodoTMT-switch LC–MS/MS) can support multiplexed quantification of candidate SNO sites, provided that thiol-blocking is complete, reduction/labeling specificity is verified, and site localization is unambiguous [66]. Within the A–C framework, these approaches support residue-level attribution when site localization is secure. Functional interpretation is strengthened only when residue assignment is accompanied by matched perturbation or rescue evidence. Where feasible, fractional-occupancy estimates add important quantitative context by helping distinguish large relative changes from shifts that may be more functionally meaningful. Residue-level mechanistic language in HF is best reserved for findings supported by Class A analytical evidence.

#### 3.2.2. Protein-Level Enrichment Approaches (Class B)

Class B workflows are most useful for comparative screening, such as identifying proteins that show condition-dependent changes in SNO enrichment across models, time windows, or interventions. Their primary support remains at the protein level. Site assignment and occupancy-related interpretation require site-resolved Class A evidence with secure localization and appropriate quantitative support where available [71,72]. Functional interpretation is strengthened when protein-level enrichment data are paired with orthogonal validation and HF-relevant functional evidence.

Biotin-Switch Technique (BST)

Across HF phenotypes, time-course, or intervention comparisons, the biotin-switch technique (BST) can be used as a front-end screen to prioritize candidate proteins showing directional changes in SNO signal. Within this framework, BST is a Class B readout. Its interpretation depends on adequate controls because incomplete thiol blocking, reductant non-specificity, and oxidative background can inflate apparent SNO signals, particularly in HF myocardium with elevated reactive oxygen species/reactive nitrogen species (ROS/RNS) flux [68,71]. Minimum controls should include a no-ascorbate control, verification of thiol-blocking efficiency, and normalization to input protein abundance. These controls make BST useful for screening, while leaving site- and occupancy-level interpretation to follow-up validation.

Resin-Assisted Capture (SNO-RAC)

Resin-assisted capture workflows (SNO-RAC) provide proteome-scale enrichment patterns that can help prioritize candidate proteins across conditions, time windows, or interventions [67]. Within this framework, SNO-RAC is best treated as a Class B readout when used primarily for enrichment analysis, because capture efficiency, resin reactivity or bias, and batch effects constrain quantitative interpretation [67,73]. SNO-RAC findings should generally be interpreted as protein-level enrichment patterns. Extension to residue-level or compartmental interpretation requires direct site localization or fractionation-aware designs with appropriate compartment markers, matched input controls, and supporting validation [61].

### 3.3. Class C: System-Level Readouts and Interpretive Limits

Class C platforms can be useful for clinical phenotyping and longitudinal assessment, but their molecular attribution remains limited. They provide broad systemic nitrosative readouts but generally cannot distinguish myocardial protein S-nitrosylation from low-molecular-weight thiol pools such as GSNO.

Photolysis–Chemiluminescence (RSNO)

For HF cohort phenotyping and longitudinal tracking, chemiluminescence-based platforms can provide sensitive systemic RSNO-related readouts. Still, they generally lack the molecular resolution required to attribute signals to myocardial protein SNO versus low-molecular-weight RSNO pools (e.g., GSNO) without paired tissue proteomics [74]. Without such pairing, these assays should remain at the systemic or biofluid-readout level.

Electrochemical RSNO/NO assay

Electrochemical platforms can provide rapid NO/RSNO-related readouts useful for cohort-level phenotyping. However, they are vulnerable to matrix interference, often cannot partition protein SNO from low-molecular-weight RSNO pools, and do not provide protein/site attribution [70]. Accordingly, these assays are best used for phenotyping and longitudinal tracking rather than for target-specific or compartment-resolved SNO remodeling.

Even beyond Class C phenotyping, interpretation remains constrained by the low abundance and chemical lability of endogenous S-nitrosothiols. In cardiac tissue, occupancy-aware datasets remain limited, and the available myocardial literature suggests that baseline occupancy can be low for some measured targets [75,76]. Relative changes in SNO signal should not be equated with high absolute occupancy. Residue-level interpretation is most defensible when secure site assignment, quantitative context, and matched functional evidence converge [76,77]. Broad human myocardial datasets with both site-level and occupancy-aware resolution remain limited, especially in HFpEF [78]. Box 1 summarizes how Analytical Class and Evidence Grade are used together in this review.

## 4. S-Nitrosylation in Heart Failure: Evidence-Graded Mechanistic Examples

This section reviews representative SNO-linked mechanisms discussed in HF-related settings. To keep interpretation proportionate to the available evidence, stronger mechanistic conclusions are reserved for observations anchored to human HF myocardium or established HF-relevant in vivo models, whereas findings derived mainly from non-HF, acute-injury, therapy-driven, or mechanistic systems are treated as context-limited or hypothesis-generating. Table 3 summarizes representative SNO events supported by human HF or HF-relevant in vivo evidence, while candidate nodes from non-HF contexts are listed separately in Supplementary Table S1. Evidence Grade (I/IIa/IIb/III) is used throughout this section to indicate disease-context relevance and biological validation depth. Where competing cysteine modifications or PTM assignments remain unresolved, interpretation is kept provisional.

### 4.1. SNO and Microvascular/Endothelial Dysfunction

Microvascular endothelial dysfunction is a prominent feature of HF, especially HFpEF, and is commonly linked to reduced NO–sGC–cGMP–PKG signaling together with inflammatory and oxidative stress [20,40,87]. By contrast, specific endothelial SNO events remain poorly defined in HF myocardium. Oxidant sources, including NADPH oxidases, are relevant here chiefly because they modify the local thiol-redox background, which can complicate interpretation of SNO-related findings [88].

Only two non-HF or context-limited endothelial SNO observations are noted here. One comes from inflammatory vascular models of atherosclerosis, in which iNOS-dependent SNO of eNOS at Cys94 and Cys99 was associated with greater eNOS–β-catenin coupling [89]. The other derives from diabetes-related models of cardiac microvascular injury, where SNO of dynamin-related protein 1 (Drp1) at Cys644 in human and Cys650 in mouse was associated with mitochondrial fragmentation and increased ferroptosis susceptibility in endothelial cells [90]. Neither observation has yet been directly validated in coronary microvascular endothelium in HF or HFpEF.

Based on current evidence, these endothelial observations are best regarded as hypothesis-generating candidate observations rather than as HF-anchored mechanisms. Direct cell-type-resolved evidence for specific endothelial SNO events in human HF, particularly in coronary microvascular endothelium, is still lacking.

### 4.2. SNO and Energetic Remodeling

Evidence linking mitochondrial or metabolic SNO to HF remains limited and uneven. To avoid conflating disease contexts, this section first considers observations from failing myocardium or HF-relevant remodeling models, and then separately summarizes acute ischemia–reperfusion, donor-based, or pharmacologic intervention studies that are less directly applicable to chronic HF.

Within HF-relevant remodeling settings, the best-anchored mitochondrial example among the studies discussed here involves GSNOR-dependent regulation of adenine nucleotide translocator 1 (ANT1) S-nitrosylation. Mitochondrial GSNOR-dependent denitrosylation of ANT1 has been linked to restored ANT1 activity and improved mitochondrial function [50].

Other findings are more restricted. ATP synthase α has been implicated in dyssynchronous HF and cardiac resynchronization studies, but current support remains limited to that setting [60]. In fibroblast-associated HF-relevant models, SNO of pyruvate kinase M2 (PKM2) has been linked to reduced enzyme activity, mitochondrial fission, and profibrotic activation [85]. This may connect metabolic remodeling with fibrosis, but it does not establish a core myocardial energetic SNO mechanism in HF.

Several additional mitochondrial SNO observations come from acute ischemia–reperfusion or pharmacologic intervention studies rather than from chronic HF remodeling. During early reperfusion in the ischemia–reperfusion model, SNO of the mitochondrial complex I NADH dehydrogenase subunit 3 (ND3) at Cys39 has been associated with reduced oxidative injury [91]. Mitochondria-targeted S-nitrosothiol delivery has also been reported to attenuate later post-infarct dysfunction/remodeling in mice, although the responsible mitochondrial cysteine target has not been defined [92]. These examples are included to mark the boundary between acute myocardial injury, donor/intervention biology, and chronic HF remodeling.

Overall, current evidence points more consistently to altered mitochondrial denitrosylation, particularly GSNOR-dependent regulation of ANT1 S-nitrosylation, than to a shared set of validated mitochondrial SNO events across HF phenotypes. Human myocardial evidence remains limited, and most mitochondrial SNO examples should therefore be interpreted as context-specific observations rather than as established energetic mechanisms in human HF.

### 4.3. SNO in Ca^2+^ Handling: HF-Relevant and Context-Limited Observations

Abnormal Ca^2+^ handling and electrical instability are well-recognized features of HF [93]. Among ECC-related SNO observations, RyR2 has relatively more direct support from an HF-relevant in vivo model. In spontaneously hypertensive HF rats, lower RyR2 S-nitrosylation was reported together with increased xanthine oxidase-derived oxidative stress, reduced free thiols consistent with greater RyR2 oxidation, and diastolic sarcoplasmic reticulum Ca^2+^ leak [59]. This rat-model evidence is HF-relevant but still limited, and it does not establish a myocardial SNO mechanism in human HF.

Other Ca^2+^-handling proteins are discussed more cautiously. Ca^2+^/calmodulin-dependent protein kinase II (CaMKII), phospholamban (PLN), and cardiac troponin C (cTnC) are discussed here because they act at related steps of Ca^2+^ release, Ca^2+^ reuptake, or myofilament response. SNO of CaMKII has been linked to Ca^2+^ leak and arrhythmias in experimental settings, but direct support in chronic HF remains limited [94]. PLN and cTnC have likewise been reported to undergo SNO in adrenergic or hypertrophy-linked settings [95]. Current evidence does not support grouping these observations into a unified SNO-based Ca^2+^-handling pathway in HF. They are included here only as examples of reported SNO events at distinct steps of Ca^2+^ handling, rather than as direct evidence for a validated mechanism in chronic HF.

Additional reports involving the *SCN5A*-encoded sodium channel Nav1.5, Kir2.1, connexin 43 (Cx43), and S100 calcium-binding protein A1 (S100A1) arise mainly from cardiomyopathy, mechanistic, or other non-HF-dominant settings [96,97,98,99]. These examples help outline the broader electrophysiology-related contexts in which SNO has been studied, but they remain context-limited with respect to chronic HF.

Overall, the evidence in this section remains uneven. Support for RyR2 is more direct than for the other proteins discussed here, but it remains limited. The remaining proteins are supported mainly by mechanistic, non-HF, or otherwise context-limited studies. Their relevance to human HF remains to be established.

### 4.4. Selected SNO-Linked Signaling Observations

Evidence for SNO-related signaling changes in HF remains uneven. A small number of stress- and remodeling-associated nodes have HF-relevant in vivo support, whereas many receptor-proximal or NO–cGMP effector examples come from non-HF, acute-injury, therapy-driven, vascular, or cell-based systems. This section therefore uses selected examples to define the current evidence boundary rather than to construct a unified SNO signaling network in HF.

Several stress- or remodeling-associated nodes have stronger HF-relevant in vivo support. SNO-linked changes involving heat shock protein 90 (HSP90), c-Jun N-terminal kinase (JNK), and muscle LIM protein (MLP) have been reported in pressure-overload or remodeling contexts and have been linked to fibrotic, hypertrophic, or inflammasome-related readouts [79,80,81,82]. Even for these nodes, direct validation in human HF myocardium and occupancy-aware site-level evidence remain limited. They are therefore best interpreted as HF-relevant in vivo observations rather than as established human HF mechanisms. Other signaling nodes, including STAT3, Akt, Keap1, and GSK3β, are discussed more cautiously because the available evidence is heterogeneous, spanning acute-stress, therapy-driven, mechanistic systems, and selected HF-relevant animal observations [100,101,102,103].

**Figure 3:**
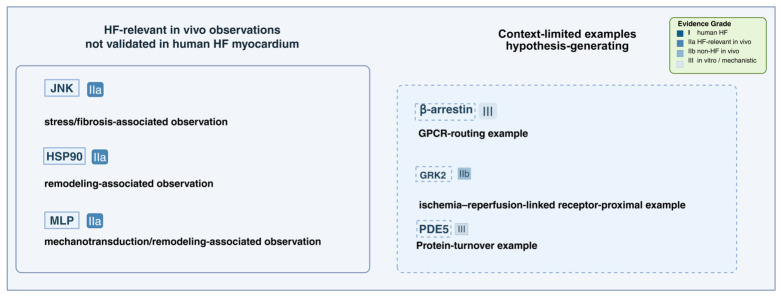
Representative SNO-linked signaling observations arranged by disease-context support. This figure summarizes selected signaling examples discussed in Section 4.4. JNK, HSP90, and MLP are shown as observations with HF-relevant in vivo support, although direct validation as mechanisms in human HF myocardium and site-resolved, occupancy-aware evidence remain limited. β-arrestin is retained as a receptor-proximal illustrative example, whereas GRK2 and PDE5 are shown as context-limited examples derived mainly from non-HF, acute-injury, therapy-driven, vascular, or cell-based studies. The inset shows the evidence-grade color scale used in this review; not all grades are necessarily represented among the displayed examples. Evidence-grade badges assigned to the displayed examples indicate the highest disease-context support used in this review: I, human HF; IIa, HF-relevant in vivo; IIb, non-HF in vivo; and III, in vitro or mechanistic. These badges should not be read as indicating analytical class, residue-level validation, or occupancy-aware evidence. Solid and dashed outlines distinguish observations with HF-relevant in vivo support from context-limited, hypothesis-generating examples. The figure is intended as a summary of representative observations, not as a validated signaling pathway or integrated signaling network in human HF. Created in BioRender. Shi, M. (2026) https://BioRender.com/3y0yr8n (accessed on 21 May 2026). Abbreviations: SNO, S-nitrosylation; HF, heart failure; JNK, c-Jun N-terminal kinase; HSP90, heat shock protein 90; MLP, muscle LIM protein; GRK2, G protein-coupled receptor kinase 2; PDE5, phosphodiesterase 5.

Receptor-proximal examples remain weakly anchored to chronic HF myocardium. In cell-based receptor-signaling models, S-nitrosylation of β-arrestins has been reported to influence receptor signaling bias and ligand responsiveness [104]. G protein-coupled receptor kinase 2 (GRK2) provides a second context-limited example. In cardiac ischemia–reperfusion settings, eNOS-dependent SNO of GRK2 has been linked to cardioprotection [105,106]. These findings are useful for illustrating how SNO may influence receptor-proximal signaling, but they have not been directly validated as chronic HF myocardial mechanisms. NO–cGMP-related effector examples require a similar level of caution. SNO of phosphodiesterase 5 (PDE5) at Cys220 has been reported in transfected cells and HL-1 cardiomyocytes to promote ubiquitin–proteasomal degradation [107]. This example indicates that SNO may modify NO-responsive effector proteins in selected experimental contexts. However, current evidence is insufficient to establish PDE5 S-nitrosylation as a myocardial signaling mechanism in chronic HF. Figure 3 summarizes representative SNO-linked observations discussed in this section. These examples vary in disease-context support and should not be interpreted as an established signaling pathway in human HF.

### 4.5. SNO in HFpEF: Current Evidence and Unresolved Questions

**Figure 4:**
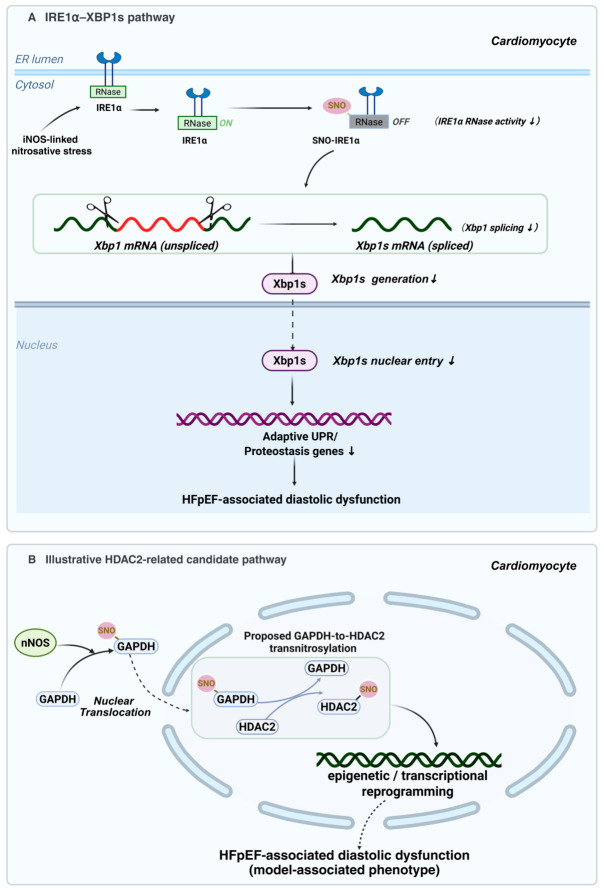
Selected SNO-linked observations in HFpEF-related settings. Panel (**A**) summarizes reported IRE1α–XBP1s-related observations, which have relatively stronger HFpEF-related disease-context anchoring than the other example shown here. Panel (**B**) illustrates a candidate GAPDH–HDAC2 SNO-related relay proposed from HFpEF-like model settings. Arrows indicate the reported or proposed direction of the candidate mechanism and should not be read as showing that every step has been directly validated in human HFpEF myocardium. Dashed arrows indicate translocation or inferred downstream phenotypic links rather than direct biochemical reactions. This figure is intended as a selective visual guide to examples discussed in Section 4.5 and does not imply equivalent evidentiary support, residue-level validation across all nodes, occupancy-aware quantification, or a validated integrated pathway in human HFpEF. Created in BioRender. Shi, M. (2026) https://BioRender.com/xug13t4 (accessed on 21 May 2026). Abbreviations: SNO, S-nitrosylation; HFpEF, heart failure with preserved ejection fraction; IRE1α, inositol-requiring enzyme 1α; XBP1s, spliced X-box binding protein 1; GAPDH, glyceraldehyde-3-phosphate dehydrogenase; HDAC2, histone deacetylase 2.

HFpEF provides an informative but still evidence-limited setting in which impaired NO–sGC–cGMP–PKG signaling may coexist with nitrosative stress [41,108]. Current data suggest that altered SNO may be relevant to selected proteostatic, metabolic, and fibro-inflammatory pathways in HFpEF-related settings. Even so, support is not uniform across nodes, and much of the available mechanistic evidence still derives from experimental models rather than from directly validated human, cysteine-resolved, occupancy-aware myocardial datasets. In this review, we do not consider HFpEF an established myocardial SNO framework. Instead, this section treats several reported SNO-linked candidate modules in HFpEF-related settings, while distinguishing more strongly anchored observations from mechanisms that remain model-supported and hypothesis-generating. Figure 4 serves as a visual guide to selected SNO-linked candidate modules discussed in this section and should not be interpreted as implying equivalent evidence depth or a fully validated integrated circuitry for human HFpEF.

Among the SNO-linked observations discussed in HFpEF-related settings, the inositol-requiring enzyme 1α (IRE1α)-spliced X-box binding protein 1 (XBP1s) pathway has the most direct disease-context anchoring. Increased myocardial SNO of IRE1α has been reported in human HFpEF myocardium, together with supporting evidence from an HFpEF mouse model, linking nitrosative stress to impaired XBP1s signaling and maladaptive proteostasis [41]. Even so, it remains uncertain how broadly this mechanism applies across distinct HFpEF endophenotypes and disease stages. By contrast, histone deacetylase 2 (HDAC2), Akt, and phosphatase and tensin homolog (PTEN) should be interpreted as more provisional observations from HFpEF-like or cell-supported settings. nNOS-dependent transnitrosylation of HDAC2 has been associated with diastolic dysfunction in HFpEF-like settings and is consistent with the possibility that nuclear SNO contributes to epigenetic remodeling [83]. Nevertheless, the disease-context anchoring of this pathway remains limited. Another pattern applies to Akt. In a murine cardiometabolic HFpEF model, increased myocardial SNO-Akt has been reported, associated with impaired Akt activation and insulin signaling [86]. However, the dominant residue assignment is primarily based on cardiomyocyte-based mechanistic studies, and residue occupancy has not been determined in HF myocardium. This module should therefore be regarded as hypothesis-generating rather than as an established myocardial SNO mechanism in HFpEF. Fibroblast-centered nitrosative signaling also remains incompletely defined in this context. An iNOS-linked PTEN–PI3K–Akt axis has been implicated in pro-fibrotic signaling in HFpEF-related experimental systems, but current evidence is insufficient to support its interpretation as a confirmed myocardial SNO mechanism in human HFpEF [84].

## 5. Clinical Interpretation and Translational Considerations Within the Limits of Current Evidence

### 5.1. Translational Premise: Beyond Pathway-Level NO Augmentation

Reduced NO–sGC–cGMP–PKG signaling does not necessarily parallel myocardial SNO remodeling. Bulk NO- or RSNO-related measures cannot, on their own, indicate which myocardial compartments or cysteine targets gain or lose SNO occupancy [40]. This point is relevant when interpreting HFpEF-related studies of long-acting organic nitrates or inhaled inorganic nitrite. Neutral or unfavorable results caution against assuming that simply increasing NO equivalents restores compartment-specific SNO signaling. However, these clinical results should not be used to validate or dismiss specific SNO-linked mechanisms without direct myocardial evidence [109,110,111]. Against this background, increasing NO equivalents alone may be insufficient without attention to compartment, target context, and denitrosylation capacity.

### 5.2. Illustrative Categories for Translational Interpretation

Under current evidence limits, it is helpful to distinguish three broad contexts for translational discussion: pathway-level NO reserve restoration, modulation of nitrosothiol pressure and denitrosylation capacity, and target- and compartment-dependent effector-level changes.

#### 5.2.1. The NO–sGC–cGMP–PKG Axis as a Comparator Pathway

Augmenting NO–sGC–cGMP–PKG signaling primarily restores a diffusible second-messenger reserve and may improve hemodynamic or relaxation buffering, but it does not necessarily normalize microdomain SNO patterns. We therefore discuss this pathway as a useful comparator: its success or failure may depend on patient selection and timing, without directly resolving the role of compartment-specific SNO changes [16].

#### 5.2.2. Interpreting Nitrosothiol Burden and Denitrosylation Capacity

Beyond NO generation, overall nitrosothiol availability is shaped by enzymes and relay systems that influence whether SNO signals remain reversible and spatially constrained. GSNOR constrains GSNO-driven transnitrosylation potential, whereas thioredoxin systems provide protein-directed denitrosylation capacity that may help preserve selectivity under oxidative stress [24]. The desired direction of denitrosylation modulation is likely to depend on phenotype, compartment, and target context. Current evidence is insufficient to support a uniform conclusion across HF-related settings.

#### 5.2.3. Effector-Level Contexts and Interpretive Boundaries

A third provisional category concerns effector-level contexts in which local SNO imbalance may map onto HF-relevant phenotypes. Because such examples are highly dependent on compartment and target context, they should be used for translational interpretation only when paired with mechanism-aligned readouts and interpreted within the evidence boundaries outlined above.

### 5.3. Biomarker and Pharmacodynamic Readout Logic

For studies that include SNO-related translational readouts, no single circulating marker is sufficient. Blood-based nitrosothiol-, nitrite-, and broader nitroso-redox measures may support phenotyping, enrichment, and serial follow-up, but they cannot by themselves establish myocardial protein-, site-, or compartment-specific target engagement [108,112]. Biomarker interpretation should therefore rely on complementary readout layers linked to mechanism-aligned functional endpoints rather than on inference from bulk NO/RSNO measures alone.

A practical framework can be organized into three readout layers. The first is a serial biofluid support layer used for enrichment, stratification, and longitudinal tracking. The second is a pathway-facing pharmacodynamic layer used to test whether the prespecified biological module shifts in the intended direction. Where feasible, this layer may be interpreted alongside cGMP/PKG-facing comparator readouts to avoid conflating pathway-level reserve effects with SNO-related readouts. The third is a tissue- or cell-informed anchor layer, ideally incorporated through mechanistic substudies, because it provides the strongest support for target engagement and keeps interpretation aligned with the attribution limits discussed in Section 3 [113].

**Figure 5:**
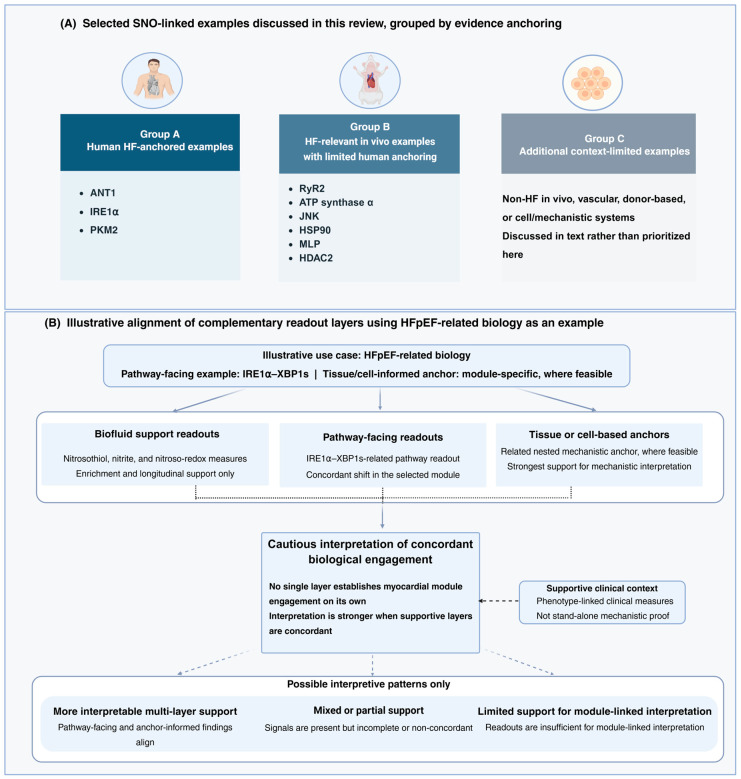
Evidence-bounded interpretation of selected SNO-linked nodes and readout layers in HF-related settings. (**A**) Selected SNO-linked examples discussed in this review are grouped according to disease-context anchoring. These display groups are derived from the Evidence Grade framework used in the text and are not intended as an additional classification system. Group A includes selected examples with human HF anchoring, corresponding broadly to Grade I evidence in this review. Group B includes examples supported mainly by HF-relevant in vivo studies with limited human anchoring, corresponding broadly to Grade IIa evidence. Group C denotes additional context-limited examples discussed in the text rather than prioritized individually in this panel. These examples are derived largely from non-HF in vivo, vascular, donor-based, cell-based/mechanistic systems and correspond broadly to Grade IIb–III evidence. The examples shown are representative and are used to illustrate differences in disease-context support rather than to provide an exhaustive inventory of all SNO-linked nodes. Grouping should not be interpreted as therapeutic priority, biomarker validation, clinical readiness, analytical class, residue-level validation, or occupancy-aware evidence. (**B**) Illustrative alignment of complementary readout layers, shown using HFpEF-related biology as an example. Biofluid support readouts, pathway-facing readouts, and tissue- or cell-informed anchor readouts are shown as complementary layers for evidence-bounded, module-linked interpretation. Clinical measures are included only as phenotype-linked supportive context and not as stand-alone mechanistic proof. Concordant findings across layers may strengthen biological interpretation, whereas mixed or incomplete readouts should remain hypothesis-generating. This panel is not intended as a therapeutic development workflow, biomarker qualification scheme, or decision algorithm. Created in BioRender. Shi, M. (2026) https://BioRender.com/a4er4w7. Abbreviations: SNO, S-nitrosylation; HF, heart failure; HFpEF, heart failure with preserved ejection fraction.

Two examples illustrate how this readout logic could be applied without implying biomarker validation. In HFpEF, the IRE1α–XBP1s module may serve as a pathway-facing proteostasis-linked readout in exploratory studies, because myocardial SNO of IRE1α has been reported in human HFpEF together with supporting evidence from an HFpEF mouse model [41]. This use should be interpreted as a mechanistic anchor for proteostasis-oriented pharmacodynamic assessment, rather than as evidence of biomarker validation. As a tissue- or cell-informed mitochondrial example, ANT1-related SNO or altered denitrosylation may be informative when myocardial sampling or cell-based mechanistic substudies are feasible, but current evidence does not support its use as a stand-alone biomarker, clinical selection marker, or prioritized therapeutic target [50]. This logic is illustrated in Figure 5, which pairs selected SNO-linked examples grouped by evidence anchoring (Figure 5A) with an illustrative framework aligning complementary readout layers in HFpEF-related biology (Figure 5B).

### 5.4. Interpreting Exploratory SNO-Related Readout Studies

At the current stage, studies involving SNO-related readouts are best framed around biological interpretability rather than efficacy claims. Under current evidence limits, the aim is not to define a universal SNO biomarker but to assess whether a prespecified biological module shows interpretable pharmacodynamic change in a biologically selected population [114,115]. Phenotype-enriched designs are therefore likely to be more informative than unselected approaches. In each case, the intended biological direction, the compartment in which engagement is expected, and the set of complementary readouts needed for interpretation should be specified in advance [113,115].

Cardiometabolic HFpEF is used here only as an illustrative use case. It provides a plausible setting in which a pathway-facing proteostasis readout, such as the IRE1α–XBP1s module, may be aligned with a phenotype-enriched study design. This should not be read as evidence that HFpEF SNO biology is fully resolved. In an exploratory study of this type, biofluid readouts may assist with enrichment and longitudinal tracking but should remain supportive rather than target-defining [108,113,116]. Evidence for biological engagement is strongest when pathway-facing pharmacodynamic readouts are supported, where feasible, by tissue- or cell-informed anchor measurements from nested mechanistic substudies [78]. Clinical endpoints should be matched to the selected phenotype but interpreted as supportive context rather than as stand-alone proof of mechanism [114,115].

At present, the practical value of such studies lies in improving interpretation of the links between exposure, biological engagement, and phenotype, rather than in implying clinical readiness.

## 6. Discussion, Evidence Boundaries, and Conclusions

Current evidence suggests that protein SNO is a context-dependent component of HF-related redox signaling rather than a uniformly adaptive or maladaptive modification. Although selected observations are anchored in human HF myocardium or HF-relevant in vivo models, many reported SNO-related findings still come from non-HF models, acute injury or reperfusion settings, pharmacologic or donor-based perturbations, vascular systems, or cell-based experiments. Such studies can help frame candidate mechanisms, but they remain insufficient for establishing causality in human HF myocardium.

This evidence gap matters because many SNO readouts still remain at the protein or system level rather than resolving specific cysteine sites or occupancy. Human myocardial datasets that combine secure site assignment, quantitative context, and matched functional validation are still scarce. For this reason, many findings are better described as protein-level changes, context-limited observations, or candidate mechanisms unless stronger analytical and disease-context validation is available.

Across HF phenotypes, current evidence does not support a simple view of SNO as globally protective or harmful. The direction and significance of a given SNO change may vary with NO source, local redox context, competing thiol modifications, subcellular compartment, and modification sequence. Current clinical data also do not support treating pathway-level NO augmentation as equivalent to correcting maladaptive SNO-linked signaling. The present review therefore separates HF-anchored observations from findings that remain provisional or hypothesis-generating. More direct human myocardial evidence, better cysteine-site resolution, quantitative or occupancy-aware information where feasible, and matched functional validation will be needed before stronger causal or translational conclusions can be drawn.

## 7. Future Directions

Future work on SNO in HF should prioritize depth over breadth. Continued expansion of candidate protein lists will be less informative than building reproducible, human-relevant evidence for a smaller number of more robustly supported candidate events.

The key issue is not simply whether a target can be detected as S-nitrosylated, but whether it shows reproducible directionality and interpretable functional relevance across models and, where possible, patient-derived material. A second priority is improved human myocardial anchoring together with better cell-type and subcellular resolution. Many current datasets still rely on homogenized myocardium and therefore cannot distinguish true redistribution of SNO signaling from changes in tissue composition. Progress will require cell-type-aware sampling, validated enrichment strategies, spatial proteomic or related high-resolution approaches, and, where feasible, occupancy-aware datasets in human HF. Claims of SNO redistribution should remain cautious unless they are supported by clear cell-type attribution or appropriate compartment-level controls [117,118,119]. A third priority is greater standardization in SNO measurement, reporting, and quantitative interpretation. Because endogenous SNO is low-abundance, labile, and highly sensitive to handling, future studies should more consistently report sample-handling safeguards, specificity controls, and the actual level of molecular attribution achieved [15,61,120]. A pragmatic path forward is to narrow from broad descriptive catalogs toward focused, well-controlled, and human-anchored SNO mechanisms, while keeping biomarker interpretation within clear attribution limits.

Only after these evidence gaps are reduced will stronger causal or translational claims become better justified.

## Figures and Tables

**Table 1:** Selected regulators and upstream inputs discussed in relation to cardiac NO/SNO handling.

Regulator/ Operational Category	Compartment/Microdomain	Main NO/SNO-Related Role	Functional Context	Readouts Used in Related Studies
nNOS (Writer)	Junctional SR/ECC microdomain	Biases local SNO of ECC targets (e.g., RyR2)	ECC/Ca^2+^ handling	SNO-RyR2 signal; Ca^2+^ spark frequency
eNOS (Writer)	Endothelial/microvascular NO niche	Sustains NO–cGMP–PKG reserve	Microvascular NO signaling/reserve	cGMP/PKG activity; pVASP (Ser239)
iNOS (Writer)	Inducible cytosol ± ER (high-flux NO)	Drives diffuse nitrosative SNO programs under inflammatory stress	Nitrosative stress remodeling	Global SNO-protein burden; 3-nitrotyrosine (3-NT)
Selected upstream nitrite-reductase inputs (context-dependent; noncanonical)				
Mb *	Cardiomyocyte cytosol/peri-mitochondrial space	Hypoxic nitrite-to-NO conversion; potential contribution to local mitochondrial NO/RSNO availability	Mitochondrial redox adaptation/hypoxia response	Nitrite-dependent mitochondrial SNO; Mb-dependent NO generation
XOR *	Cytosol/endothelial-interstitial interface	Low-O_2_/acidic nitrite-to-NO conversion; SNO flux support	Ischemia-linked redox balance/perfusion reserve	XOR-dependent nitrite-to-NO activity; nitrite-responsive NO bioavailability
SCAN (Relay)	Mitochondria–cytosol interface (SNO-CoA pool)	Routes SNO-CoA-derived nitrosylating equivalents to protein thiols	Metabolic SNO routing (HF relevance under investigation)	SNO-CoA/CoA ratio; SNO-CoA-dependent target SNO-protein
GAPDH (Relay)	Cytosol → nucleus (stress-responsive shuttling)	Couples cytosolic SNO signals to nuclear transnitrosylation and transcription	Stress-responsive transcriptional signaling	SNO-GAPDH; nuclear SNO-protein
GSNOR (Eraser)	Cytosol (GSNO pool control)	Constrains GSNO levels and GSNO-dependent transnitrosylation	RSNO pool control/remodeling context	GSNO concentration; GSNOR activity
Trx/TrxR (Eraser)	Cytosol and mitochondria (denitrosylation hubs)	Edits and terminates protein-SNO to restore reduced thiols	Redox homeostasis/stress susceptibility	Trx activity; denitrosylation capacity
SCoR (Eraser)	Mitochondria (SNO-CoA/CoA pool)	Reduces SNO-CoA and regenerates CoA, shaping carrier availability	Mitochondrial energetics	SNO-CoA level; SCoR activity

Regulators are grouped as writers, relays, and erasers to summarize source input, transnitrosylation routing, and denitrosylation. This grouping should not be read as assigning equivalent evidence depth or causal weight to all nodes. In addition to the canonical cardiac NOS isoforms, selected context-dependent nitrite-reductase inputs are included because they may contribute to local NO/RSNO availability under hypoxic, ischemic, or circulatory conditions. These entries should not be interpreted as established chronic cardiac SNO writers equivalent to compartment-resolved NOS signaling. “Functional context” indicates the domain in which each regulator or input is discussed in this review and is intended as a classification aid rather than evidence of a causal HF mechanism. “Readouts used in related studies” lists representative target- or pathway-level measures reported in cardiac or HF-relevant studies; these readouts do not necessarily provide site-resolved or occupancy-aware SNO attribution. Where site-resolved PTM discrimination is lacking, SNO-related interpretations should be considered alongside competing cysteine modifications. * Mb and XOR are included as conditional upstream nitrite-reductase inputs that may influence local NO/RSNO availability under low-O_2_, ischemic, or redox-stressed conditions. They are not classified here as canonical NOS writers or direct, site-selective SNO relay enzymes. Mb denotes myoglobin; in this row, nitrite-reductase activity refers specifically to deoxygenated myoglobin (deoxyMb). Abbreviations: NO, nitric oxide; SNO, S-nitrosylation; RSNO, S-nitrosothiol; NOS, nitric oxide synthase; nNOS, neuronal nitric oxide synthase; eNOS, endothelial nitric oxide synthase; iNOS, inducible nitric oxide synthase; ECC, excitation–contraction coupling; SR, sarcoplasmic reticulum; ER, endoplasmic reticulum; PKG, protein kinase G; pVASP, phosphorylated vasodilator-stimulated phosphoprotein; Mb, myoglobin; XOR, xanthine oxidoreductase; SCAN, SNO-CoA-assisted nitrosylase; GAPDH, glyceraldehyde-3-phosphate dehydrogenase; GSNOR, S-nitrosoglutathione reductase; Trx/TrxR, thioredoxin/thioredoxin reductase; SCoR, SNO-CoA reductase; GSNO, S-nitrosoglutathione; CoA, coenzyme A; SNO-CoA, S-nitrosylated coenzyme A; 3-NT, 3-nitrotyrosine. cGMP, cyclic guanosine monophosphate; RyR2, ryanodine receptor 2.

**Table 2:** Analytical classes and corresponding writing ceilings for interpreting S-nitrosylation studies in heart failure.

Analytical Class	Method	Major Limitation	Writing Ceiling	Refs
A	IodoTMT-switch LC–MS/MS	High sample demand; complex normalization	Residue-level attribution; occupancy-aware interpretation where feasible	[65,66]
B	Resin-Assisted Capture (SNO-RAC)	Resin bias; inter-batch variability	Protein-level enrichment; avoid residue- or occupancy-level claims	[67]
	Biotin-Switch Technique (BST)	Non-specific reduction; semi-quantitative	Directional protein-level SNO signal; avoid residue- or occupancy-level language	[68]
C	Photolysis–Chemiluminescence (RSNO)	No molecular specificity; interpretive ambiguity	Systemic RSNO index; no protein attribution	[69]
	Electrochemical RSNO/NO assay	Matrix interference; lacks protein specificity	Rapid NO/RSNO-related readout; phenotyping or association-level use only	[70]

Analytical classes (A–C) are assigned according to the highest level of molecular attribution each workflow can typically support: residue level, protein level, or system level. These classes are intended to define attribution limits rather than to rank the overall quality or utility of individual methods. “Writing ceiling” indicates the most specific wording justified by that readout. Class C methods may assist phenotyping or longitudinal assessment, but they do not, by themselves, support inference about specific myocardial protein targets or cysteine sites. Residue-level language in HF should be reserved for Class A datasets with secure site localization, whereas occupancy-aware wording requires appropriate quantitative design and support. Abbreviations: BST, biotin-switch technique; HF, heart failure; iodoTMT, iodo-tandem mass tag; LC–MS/MS, liquid chromatography–tandem mass spectrometry; MS, mass spectrometry; NO, nitric oxide; RSNO, S-nitrosothiol; SNO, S-nitrosylation; SNO-RAC, resin-assisted capture; TMT, tandem mass tag.

**Box 1:** How Analytical Class and Evidence Grade are used in this review.

**Axis**	**What It Evaluates**	**What It Does Not Establish**
Analytical Class (A–C)	The level of attribution a readout can support: system level, protein level, or residue level, and, in some studies, limited occupancy-aware interpretation.	Disease-context relevance, clinical validation, or therapeutic readiness.
Evidence Grade (I, IIa, IIb, III)	The level of HF-context anchoring, ranging from human HF myocardium to HF-relevant in vivo models, non-HF in vivo models, and in vitro or mechanistic systems.	Site localization, occupancy, or analytical specificity.
In this review, stronger mechanistic wording is reserved for findings in which adequate molecular attribution and HF-context validation converge. When either axis is incomplete, findings are described as protein-level changes, system-level associations, context-limited observations, or hypothesis-generating mechanisms rather than as established HF mechanisms.		

**Table 3:** Summary of representative S-nitrosylation (SNO) events linked to HF-relevant mechanistic modules.

Target Protein	SNO Site	HF Context/Model	ΔSNO (HF)	HF Module Tag	Key HF-Relevant Readout	Evidence Grade	Ref
ANT1	C160	Human HF (failing myocardium) + HF-relevant in vivo (TAC)	**↑**	Mitochondrial energetics	Impaired OXPHOS (Δψm ↓); defective mitophagy	I	[50]
ATP synthase α	C294	Canine dyssynchronous HF/CRT model	**↓**	Mitochondrial energetics	ATP synthase activity recovery; improved energetic efficiency	IIa	[60]
HSP90	C589	Human non-HF (valve disease) + HF-relevant in vivo (TAC fibrosis)	**↑**	Fibrosis/remodeling	TGFβ–SMAD3 activation; fibrosis progression	IIa	[79]
HSP90	C589	HF-relevant in vivo (TAC pressure overload)	**↑**	Hypertrophy/remodeling	GSK3β signaling reprogramming; hypertrophic remodeling	IIa	[80]
JNK	C116, C163	Human non-HF (valve disease ± hypertrophy) + HF-relevant in vivo (TAC pressure overload)	**↑**	Fibrosis/stress signaling	AP-1 activation; fibrosis progression	IIa	[81]
MLP	C79	Human non-HF (valve disease ± hypertrophy) + HF-relevant in vivo (TAC hypertrophy)	**↑**	Fibro-inflammatory remodeling	TLR3–NLRP3 inflammasome activation; hypertrophic remodeling	IIa	[82]
IRE1α	NR	Human HF (HFpEF myocardium) + HFpEF mouse (HFD + L-NAME)	**↑**	Proteostasis/ER stress	XBP1s deficiency; maladaptive UPR signaling	I	[41]
HDAC2	C262, C274	Human non-HF (LVH) + HFpEF-like DD mouse (SAUNA/mTAC)	**↑**	Epigenetic/stiffness control	Diastolic dysfunction; transcriptional reprogramming	IIa	[83]
PTEN	NR	HFpEF mouse (HFD + L-NAME) + in vitro (cardiac fibroblasts)	**↑**	Fibrosis	PTEN protein ↓; p-PI3K/p-Akt ↑; collagen I/III ↑; fibroblast proliferation/migration ↑	IIa ‡	[84]
PKM2	C49, C326	Human HF (failing myocardium) + HF-relevant in vivo (TAC fibrosis)	**↑**	Metabolic–fibrotic remodeling	Myofibroblast differentiation PKM2 activity ↓	I	[85]
RyR2	NR	HF-relevant in vivo (SHHF rat dilated cardiomyopathy HF)	**↓**	ECC/Ca^2+^ handling	Diastolic SR Ca^2+^ leak; Ca^2+^-handling instability	IIa	[59]
Akt	C224 †	HFpEF mouse (HFD + L-NAME) + in vitro (cardiomyocytes)	**↑**	Metabolic stress/insulin signaling	Akt signaling (p-Akt Ser473) ↓; glucose uptake ↓	IIa	[86]

Table 3 summarizes S-nitrosylation (SNO) events that have been directly measured in heart failure (HF) myocardium or in established HF-relevant in vivo models and links each target to core HF pathophysiological modules. Evidence Grade reflects disease-context relevance and biological validation depth: Grade I, human HF myocardium; Grade IIa, established HF-relevant animal models (e.g., TAC, HFpEF); Grade IIb, in vivo animal models not directly modeling HF; and Grade III, in vitro or cell-based systems only. SNO directionality (↑/↓) refers to changes observed within the specified HF context, rather than manipulation-driven or pharmacological perturbations unless explicitly stated. For targets with residue-level information, reported cysteine sites indicate either direct in vivo mapping or mechanistic site assignment supported by cellular mutagenesis, as specified in footnotes. ‡ For PTEN, increased SNO-PTEN was detected at the protein level in HFD + L-NAME HFpEF mouse left ventricle, with supporting cardiac fibroblast experiments. Site localization, fractional occupancy, and in vivo PTEN-specific causal validation were not performed. † For Akt, increased SNO-Akt was detected at the protein level in HFD + L-NAME HFpEF mouse hearts, whereas Cys224 assignment was supported by cardiomyocyte/AC16 mutagenesis assays. Residue-level mapping and occupancy were not determined in HFpEF myocardium. NR, not resolved/not reported at residue level. Evidence Grade denotes disease-context anchoring only and does not imply residue-level mapping, occupancy-aware quantification, or therapeutic readiness. Analytical attribution is limited by the underlying method and is indicated by site notation and footnotes where relevant. For entries in which the HF-context readout is protein-level or pathway-level, the listed cysteine site should be interpreted only as a mechanistic site assignment from supporting experiments and not as site-resolved validation in HF myocardium. Abbreviations: ANT1, adenine nucleotide translocator 1; HSP90, heat shock protein 90; TGFβ, transforming growth factor beta; SMAD3, SMAD family member 3; GSK3β, glycogen synthase kinase 3β; JNK, c-Jun N-terminal kinase; MLP, muscle LIM protein; TLR3, Toll-like receptor 3; NLRP3, NLR family pyrin domain-containing 3; IRE1α, inositol-requiring enzyme 1α; XBP1s, spliced X-box binding protein 1; HDAC2, histone deacetylase 2; PTEN, phosphatase and tensin homolog; PI3K, phosphoinositide 3-kinase; Akt, protein kinase B; p-Akt, phosphorylated Akt; PKM2, pyruvate kinase M2; RyR2, ryanodine receptor 2; Δψm, mitochondrial membrane potential. AP-1, activator protein 1; CRT, cardiac resynchronization therapy; DD, diastolic dysfunction; HFD, high-fat diet; L-NAME, Nω-nitro-L-arginine methyl ester; LVH, left ventricular hypertrophy; NR, not resolved/not reported at residue level; OXPHOS, oxidative phosphorylation; SHHF, spontaneously hypertensive heart failure; TAC, transverse aortic constriction; UPR, unfolded protein response. Residue-level language in this table reflects the maximum attribution supported by the underlying analytical class and should not be extrapolated beyond the specified HF context.

## Data Availability

No new data were created or analyzed in this study. Data sharing is not applicable to this article.

## Supplementary Materials

The following supporting information can be downloaded at: https://www.mdpi.com/article/10.3390/antiox15060716/s1, Table S1: Context-limited S-nitrosylation nodes for HF-focused hypothesis generation.

## Abbreviations

The following abbreviations are used in this manuscript. Figure- and table-specific abbreviations are defined in the corresponding captions and footnotes.

ANT1	adenine nucleotide translocator 1
BST	biotin-switch technique
cGMP	cyclic guanosine monophosphate
CoA	coenzyme A
Cys	cysteine
ECC	excitation–contraction coupling
eNOS	endothelial nitric oxide synthase
ER	endoplasmic reticulum
GAPDH	glyceraldehyde-3-phosphate dehydrogenase
GPCR	G protein-coupled receptor
GRK2	G protein-coupled receptor kinase 2
GSH	glutathione
GSK3β	glycogen synthase kinase 3β
GSNO	S-nitrosoglutathione
GSNOR	S-nitrosoglutathione reductase
HDAC2	histone deacetylase 2
HF	heart failure
HFD	high-fat diet
HFmrEF	heart failure with mildly reduced ejection fraction
HFpEF	heart failure with preserved ejection fraction
HFrEF	heart failure with reduced ejection fraction
HSP90	heat shock protein 90
iNOS	inducible nitric oxide synthase
iodoTMT	iodo-tandem mass tag
IRE1α	inositol-requiring enzyme 1α
JNK	c-Jun N-terminal kinase
L-NAME	Nω-nitro-L-arginine methyl ester
LC–MS/MS	liquid chromatography–tandem mass spectrometry
MLP	muscle LIM protein
MS	mass spectrometry
NAD(P)H	reduced nicotinamide adenine dinucleotide or reduced nicotinamide adenine dinucleotide phosphate
nNOS	neuronal nitric oxide synthase
NO	nitric oxide
NOS	nitric oxide synthase
PDE5	phosphodiesterase 5
PKG	protein kinase G
PKM2	pyruvate kinase M2
PTEN	phosphatase and tensin homolog
PTM	post-translational modification
RNS	reactive nitrogen species
ROS	reactive oxygen species
RSNO	S-nitrosothiol
RyR2	ryanodine receptor 2
SCAN	SNO-CoA-assisted nitrosylase
SCoR	SNO-CoA reductase
sGC	soluble guanylate cyclase
SNO	S-nitrosylation
SNO-CoA	S-nitrosylated coenzyme A
SNO-RAC	resin-assisted capture for S-nitrosylated proteins
SR	sarcoplasmic reticulum
TAC	transverse aortic constriction
Trx	thioredoxin
TrxR	thioredoxin reductase
UPR	unfolded protein response
XBP1s	spliced X-box binding protein 1
XOR	xanthine oxidoreductase
