# Supplementary material for: Protein S-Nitrosylation in Heart Failure: A Compartment-Resolved Review of Mechanisms, Evidence Boundaries, and Translational Perspectives
Source: Antioxidants (Basel). 2026 Jun 4;15(6):716. doi: 10.3390/antiox15060716 (PMC13295539; doi:10.3390/antiox15060716)
Supplement: Supplementary file 1 [file antioxidants-15-00716-s001.zip › antioxidants-4166373-supplementary.pdf]

antioxidants

MDPI

Supplementary Table S1. Context-limited S-nitrosylation nodes for HF-focused hypothesis generation

Target protein	SNO site	Primary context (non-HF / non-direct HF)	Reported direction	Inference flag	Putative HF module linkage	Key readout used in source	Evidence	Ref
Drp1	Human C644 Mouse C650	Diabetic cardiomyopathy; microvascular injury	↑	CTX-nonHF	Microvascular injury / energetics	Endothelial mitochondrial fission; ferroptosis susceptibility	IIb	[90]
ND3 subunit	C39	Ischemia-reperfusion model MitoSNO intervention	↑ (manipulation-based)	MANIP; CTX-nonHF	Mitochondrial ROS control	Complex I "gating"; ROS↓ early reperfusion	IIb	[91]
cTnC	C84	Manipulation-based / adrenergic-hypertrophy context	↑ (manipulation-based)	MANIP; CTX-nonHF	ECC / myofilament	Ca^{2+} sensitivity↓	IIb	[95]
PLN	C36, C41	Adrenergic/hypertrophy context; β-adrenergic signaling models	↑ (manipulation-based)	CTX-nonHF; FUNC-extrap; MANIP SITE-inferred/mutation-supported	ECC / Ca^{2+} handling	PLN pentamerization; SERCA2a activation; SR Ca^{2+} load↑	IIb	[95]
Cx43	C271	Dystrophic cardiomyopathy; stress arrhythmia model	↑	CTX-nonHF; FUNC-extrap	ECC / electrophysiology	Hemichannel opening; arrhythmias	IIb	[98]
STAT3	C259	Myocardial ischemia–reperfusion; Stc1/CaSR/NOS2 signaling	↓ (manipulation-based)	CTX-nonHF; MANIP; SITE-inferred/mutation-supported	Stress signaling / remodeling	Ser727 phosphorylation gating; cardiomyocyte apoptosis/pyroptosis; neutrophil polarization	IIb	[100]

GRK2	C340	β -adrenergic receptor signaling; cardiac ischemia–reperfusion	↑ (eNOS/GSNO-dependent)	CTX-nonHF; MANIP; FUNC-extrap	GPCR routing	β -adrenergic desensitization restraint; I/R injury outcome	IIb	[105]
PDE5	C220	Transfected PDE5 systems; HL-1 cardiomyocyte-line NO-donor models	↑ (NO donor-induced); activity/abunda	CTX-nonHF; MANIP; FUNC-extrap	NO–cGMP–PKG / protein turnover	PDE5 ubiquitination and proteasomal degradation; PDE5	III	[107]

Supplementary Table S1 summarizes reported protein S-nitrosylation (SNO) events identified in context-limited experimental systems that do not directly constitute heart failure (HF) models. These entries are included to provide a hypothesis-generating landscape of SNO-sensitive proteins that intersect conceptually with HF-relevant functional modules (e.g., mitochondrial energetics, excitation–contraction coupling, GPCR signaling), but do not represent established HF mechanisms. For each protein, the table reports the reported or supported SNO site(s), the primary experimental context, the reported direction of SNO change **and, where relevant, associated functional consequences**, inference flags indicating contextual or mechanistic limitations, the putative HF module linkage (aligned with the module taxonomy used in Table 3), and the key functional readout used in the source study. Evidence level reflects biological validation depth and disease-context relevance, following the grading scheme defined in the main text. Importantly, inclusion in Supplementary Table S1 does not imply HF causality or confirmed disease relevance, and these nodes should be interpreted as candidates for future HF-focused validation rather than confirmed components of HF pathophysiology.

Evidence Grade definition: IIb: In vivo models not explicitly constituting HF; III: In vitro or mechanistic systems only (Evidence grading reflects biological validation depth and HF disease-context relevance, and is orthogonal to analytical resolution of SNO detection.)

Inference flags:

CTX-nonHF: Experimental context is non-HF or non-direct HF, including acute-injury, cardiometabolic, dystrophic, vascular, or mechanistic signaling systems.

MANIP: SNO directionality or functional effects derived primarily from experimental manipulation (e.g., NO donors, overexpression, mutagenesis, pharmacologic induction).

SITE-inferred: Site-specific SNO assignment or direction inferred from indirect or mechanistic evidence rather than direct site-resolved measurement.

FUNC-extrap: Functional relevance extrapolated from acute injury or non-HF contexts rather than chronic HF remodeling.

Reported direction : ↑ / ↓ indicate relative changes in SNO signal or functional consequence reported in the original study. (inferred) denotes directionality deduced from mechanistic reasoning rather than direct quantitative comparison.

https://doi.org10.3390/antiox15060716

Putative HF module linkage: Module linkage denotes conceptual intersection with HF-relevant functional domains (e.g., mitochondrial energetics, ECC, GPCR routing), using the same module taxonomy as in Table 3. These linkages are intended for conceptual integration and do not imply demonstrated involvement in HF pathophysiology. **Residue-level language in this table reflects the maximum attribution supported by the underlying analytical class and should not be extrapolated beyond the specified experimental context.**

https://doi.org10.3390/antiox15060716
